# Efficacy and safety of CD22 chimeric antigen receptor (CAR) T cell therapy in patients with B cell malignancies: a protocol for a systematic review and meta-analysis

**DOI:** 10.1186/s13643-021-01588-7

**Published:** 2021-01-21

**Authors:** Komal Adeel, Nathan J. Fergusson, Risa Shorr, Harold Atkins, Kevin A. Hay

**Affiliations:** 1 Faculty of Medicine, University of British Columbia, Vancouver, BC Canada; 2 Department of Medicine, Schulich School of Medicine and Dentistry, Western University, London, ON Canada; 3 The Ottawa Hospital Research Institute, Ottawa, Ontario Canada; 4 The Ottawa Hospital, Health Professions Education, Ottawa, Ontario Canada; 5 Faculty of Medicine, University of Ottawa, Ottawa, Canada; 6 Blood and Marrow Transplant Program, The Ottawa Hospital, Ottawa, Ontario Canada; 7 Terry Fox Laboratory, BC Cancer Research Centre, 675 West 10th Ave, Vancouver, BC V5Z 1 L3 Canada; 8 L/BMT Program of BC, Vancouver, BC Canada

**Keywords:** Chimeric antigen receptor, CAR T cell, CD22, B cell malignancies, Complete response, Adverse events, Efficacy, Safety

## Abstract

**Background:**

Chimeric antigen receptor (CAR) T cell therapy has had great success in treating patients with relapsed or refractory B cell malignancies, with CD19-targeting therapies now approved in many countries. However, a subset of patients fails to respond or relapse after CD19 CAR T cell therapy, in part due to antigen loss, which has prompted the search for alternative antigen targets. CD22 is another antigen found on the surface of B cells. CARs targeting CD22 alone or in combination with other antigens have been investigated in several pre-clinical and clinical trials.

Given the heterogeneity and small size of CAR T cell therapy clinical trials, systematic reviews are needed to evaluate their efficacy and safety. Here, we propose a systematic review of CAR T cell therapies targeting CD22, alone or in combination with other antigen targets, in B cell malignancies.

**Methods:**

We will perform a systematic search of EMBASE, MEDLINE, Web of Science, Cochrane Register of Controlled Trials, clinicaltrials.gov, and the International Clinical Trials Registry Platform. Ongoing and completed clinical trials will be identified and cataloged. Interventional studies investigating CD22 CAR T cells, including various multi-antigen targeting approaches, in patients with relapsed or refractory B cell malignancies will be eligible for inclusion. Only full-text articles, conference abstracts, letters, and case reports will be considered. Our primary outcome will be a complete response, defined as absence of detectable cancer. Secondary outcomes will include adverse events, overall response, minimal residual disease, and relapse, among others. Quality assessment will be performed using a modified Institute of Health Economics tool designed for interventional single-arm studies. We will report a narrative synthesis of clinical studies, presented in tabular format. If appropriate, a meta-analysis will be performed using a random effects model to synthesize results.

**Discussion:**

The results of the proposed review will help inform clinicians, patients, and other stakeholders of the risks and benefits of CD22 CAR T cell therapies. It will identify gaps or inconsistencies in outcome reporting and help to guide future clinical trials investigating CAR T cells.

**Systematic review registration:**

PROSPERO registration number: CRD42020193027

**Supplementary Information:**

The online version contains supplementary material available at 10.1186/s13643-021-01588-7.

## Background

Chimeric antigen receptor (CAR) T cell therapy is an immunotherapy in which T cells are genetically engineered to express CARs that target specific tumor-associated antigens, thus redirecting T cell effector function towards the malignant cells. CAR T cell therapies targeting CD19 have shown significant efficacy in treating patients with relapsed or refractory (r/r) B cell malignancies, with clinical trials of CD19 CAR T cells showing complete remission rates of 70–90% in B cell acute lymphoblastic leukemia (B-ALL) [1–3] and 40–58% in Non-Hodgkin’s Lymphoma (NHL) [4]. Given the excellent outcomes with this therapy in patients who previously had no curative option, CD19 CAR T cell therapies are now approved in many countries worldwide for the treatment of r/r B-ALL and diffuse large B cell lymphoma (DLBCL). However, around 30% of patients fail to respond to this therapy [5], and approximately 30% of patients who achieve complete remission later relapse [6, 7]. Relapse is in part attributed to CD19 antigen loss on malignant cells [8], which has prompted a search for alternative antigen targets.

CD22, like CD19, is an antigen that is restricted to the B cell lineage and as such is an ideal target for CAR T cell therapy [9]. CD22 CAR T cells have been investigated for the treatment of B cell malignancies in several pre-clinical and clinical trials, particularly in the setting of relapse after CD19 CAR T cells [10, 11]. Additionally, one potential approach under investigation to prevent antigen-escape relapse is targeting multiple antigens simultaneously [12]. As such, CAR T cell therapies that target both CD19 and CD22 in combination are being investigated and have entered clinical trials [13–17].

Preliminary results from CD22 CAR T cell therapy trials are promising, demonstrating non-trivial complete remission rates and relapse-free survival in patients with treatment-refractory disease. However, CAR T cell therapies are known to carry a risk of serious and even lethal adverse events, such as cytokine release syndrome [18] and neurotoxicity [19], making it critical to evaluate the safety of these new therapies.

Given the heterogeneity and small size of CAR T cell therapy clinical trials, systematic reviews are essential to evaluate their efficacy and safety. In preparation for this review, we conducted a scoping search of CAR T cell systematic reviews. We identified several systematic reviews focused on safety and efficacy of CD19 CAR T cell therapies [20–22]. Furthermore, Grigor et al. and Yu et al. conducted systematic reviews of all CAR T cell therapies [5, 23]. These reviews only included studies up to late 2017 and early 2018, respectively, and both studies excluded conference abstracts. Given that the first full-text clinical report of CD22 CAR T cell therapy was published in November 2017 [24], with several other studies subsequently being published [11, 14, 15, 25], these reviews do not capture the majority of currently available evidence on CD22 CAR T cell therapy. A systematic review of CD22 CAR T cells is therefore lacking in the literature. Here, we propose a systematic review of the efficacy and safety of CD22 CAR T cell therapies, alone or in combination with other antigen targets, for B cell malignancies.

## Methods

### Eligibility criteria

Eligibility criteria are presented in Table 1 in the Appendix. The population of interest will include patients of any age with B cell malignancies. Interventional studies on CAR T cell therapy targeting CD22, with and without a comparator, will be considered; the majority are expected to be single-arm interventional studies.

Study outcomes reported in full-length articles, conference abstracts, letters, and case reports will be considered for inclusion. Studies which fail to describe the CAR target or do not provide any of the clinical outcomes of interest will be excluded. Additionally, reports that cannot be connected to a registered clinical trial will be excluded. We will attempt to cross-reference the study name and authors listed in the report, and contact corresponding authors, to identify the associated clinical trial number. If the article states that the patient was treated outside of a trial for compassionate use, this is acceptable. Reviews, editorials, and commentaries will be excluded.

### Information sources

We will search MEDLINE, EMBASE, Web of Science, and the Cochrane Central Register of Controlled Trials from inception to July 8, 2020. To be comprehensive, we will examine reference lists of included studies or relevant reviews identified through the search. We will also search directly the conference proceedings of the American Society of Hematology, American Society of Clinical Oncology, and European Hematology Association, and include conference abstracts not captured by the initial search. When needed, we will search for published clinical trial protocols or trial registries to supplement information. Authors of the studies included in the review will be contacted if needed, to clarify reported outcomes.

ClinicalTrials.gov and the WHO International Clinical Trials Registry Platform (ICTRP) will be searched to identify and catalog ongoing or recently completed trials.

### Search strategy

The search strategy will be created in collaboration with a Health Science Librarian (HSL) with expertise in systematic reviews. Furthermore, the strategy will be peer reviewed by a second HSL not associated with the review. Keywords related to CAR T cell therapy and CD22 will be used. After the search is finalized in EMBASE, the subject headings and syntax will be adapted to the other databases. A draft of the search strategy can be found in the supplementary materials. No time restriction will be applied. Publications in all languages will be considered, and any non-English/French publications will be translated using Google Translate to determine eligibility.

### Data extraction (selection and coding)

The literature search results will be uploaded to Covidence, a screening and data extraction tool recommended by Cochrane. After duplicates are removed from the search results, two reviewers will independently perform a title and abstract screen using the abovementioned eligibility criteria. For titles and abstracts that meet the criteria, and those for which there is uncertainty, the full publication will be accessed. Two reviewers will then review the full publications for eligibility. Disagreements will be resolved by a third review author.

Multiple reports of the same study will be grouped. The main source of study data will be the most recent full-text journal article which provides the primary outcome and bulk of secondary outcome data. Additional reports that provide supplementary outcome information will be clearly referenced. Any additional reports that do not provide any novel information of interest will be excluded as duplicates. If a study does not have a full-text journal article which meets these criteria, then, the most recent and complete report of the primary outcomes will be used as the main source of study data.

For non-English/French publications, Google Translate will be used to convert text into English for data extraction.

### Data items

Data will be extracted in duplicate for the following variables (adapted from Grigor et al. [26]):

- Publication characteristics: title, publication type, first author, clinical trial registration number, financial support
- Study characteristics: trial design, recruitment/sampling method, inclusion criteria, sample size
- Patient characteristics: mean age, sex, malignancy diagnosis and status (ex. relapse/refractory), previous treatments (ablative, transplant, CAR T cell therapy), comorbidities, absolute lymphocyte count prior to intervention, blast levels prior to intervention, concomitant medications, and length of follow-up
- Intervention characteristics:
  - Lymphodepletion method
  - CAR T cell characteristics: T cell origin (autologous vs. allogeneic), selection of T cell subsets, T cell expansion method, fresh vs. frozen, CAR target antigen, CAR molecular structure (costimulatory domains; multi-antigen vs. single antigen)
  - Transfection/transduction method and the therapeutic regimen (CAR T cell dose, frequency, duration, route of administration).
- Outcomes:
  - Measures of efficacy: complete response, overall response, minimal residual disease (for B-ALL), progressive disease, relapse, overall survival, progression-free survival, B cell aplasia, CAR T cell expansion and persistence, stem cell transplant post CAR T cell therapy
  - Antigen expression (CD19, CD22) on malignant cells before and after CAR T cell therapy
  - Adverse events (cytokine release syndrome, neurotoxicity, infection, graft-versus-host disease)
  - Manufacturing outcomes
  - Quality of life and patient-reported outcomes

Data extraction will be recorded via a piloted data extraction form on Covidence. Disagreements between reviewers will be resolved first by discussion and then if necessary by a third-party reviewer. Where necessary, study authors will be contacted for additional data or clarifying information. An intention to treat analysis will be used when applicable.

### Outcomes and prioritizations

#### Primary outcome

Complete response (CR), defined as absence of detectable cancer, will be our primary outcome. For all reports with *N* > 1, we will record the proportion of patients who achieved a complete response at 1 month and/or at 3 months, depending on the type of data reported. If available, we will also report “best CR rate,” defined as the proportion of patients who achieved CR at any point during follow-up.

If complete response is not reported, we will report secondary response outcomes, using overall response when available. Any study which recruited patients in complete remission will be excluded from complete response data reported. We will record the criteria used within each study to define complete response and overall response.

#### Secondary outcomes

Overall response, minimal residual disease, progressive disease, relapse, overall survival, progression-free survival, B cell aplasia, CAR T cell expansion and persistence, antigen expression on malignant cells, bridging to stem cell therapy post-CAR T cell transplant, adverse events, and manufacturing outcomes are our secondary response outcomes to be measured. For all reports with *N* > 1, the proportion of patients with each outcome will be recorded (ex. overall response rate, proportion of patients with minimal residual disease) as reported in the publication.

- *Overall response*: we will define overall response as the sum of complete responses and partial responses (objective response to therapy but does not meet criteria for complete response).
- *MRD* (*minimal residual disease*): we will define minimal residual disease as the presence of leukemic cells at levels that are below the detection threshold of standard morphological assays. MRD-negative remission is an important measure of treatment response as well as a prognostic factor for relapse. When available, MRD-negative complete remission rates and MRD-positive complete remission rates will be recorded. We will also record the assay type used to detect MRD (ex. flow cytometry, RQ-PCR, NGS) and the assay’s limit of detection.
  - Note: MRD is only used widely for leukemia; therefore, this variable will not be assessed for patients with NHL.
- *Progressive disease*: we will define progressive disease as evidence of disease increases in the peripheral blood or bone marrow, or progressive or new extramedullary disease. Stable disease is defined as not meeting criteria for partial response, complete response, or progression.
- *Relapse*: we will define relapse as a partial or complete response but subsequent disease progression. Studies that recruit patients in complete remission at the initiation of CAR T cell therapy will be descriptively reported in the proportion of the patients that relapse.
- *Overall survival*: we will define overall survival as the time from the start of treatment to the time of death from any cause. If available, we will also report overall survival rate at 6 months.
- *Progression-free survival:* we will define progression-free survival as the time from the start of treatment to time of disease progression.
- *B cell aplasia*: B cell aplasia, defined as the depletion or absence of B cells, will be evaluated for its ability to predict treatment efficacy and persistence. As with CD19, CD22 is expressed on normal B cell lymphocytes. B cell aplasia is therefore an expected outcome with these CAR T cell therapies. In CD19 CAR T cell trials, B cell aplasia has been shown to be a treatable and tolerable toxicity. Furthermore, B cell aplasia appears to be a marker of CAR T cell efficacy and persistence, and thus an indicator of treatment success and risk of relapse [27].
- *CAR T cell expansion and persistence*: CAR T cell expansion and persistence is an important measure of treatment efficacy and has been shown to correlate with risk of early, antigen-positive relapse [1, 6, 28, 29]. We will report CAR T cell count in the peripheral blood as measured by flow cytometry or PCR.
- Stem cell transplant post-CAR therapy: hematopoietic stem cell transplant (HSCT) is a curative treatment for hematological malignancies, but only patients in complete remission are eligible for HSCT. One question being explored is whether CAR T cell therapy can provide a “window of opportunity” by allowing patients to achieve CR, and whether it is optimal to bridge these patients to HSCT [25, 30]. For this reason, we will report the proportion of patients achieving CR who are bridged to HSCT. If available, we will further break down this outcome and record the proportion of patients eligible for HSCT post-CAR T cell therapy and the proportion of patients who actually received HSCT, to better identify barriers in receiving HSCT.
- *Antigen expression* (*CD19, CD22*) *on malignant cells before and after CAR T cell therapy*: an established mechanism of relapse post-CAR T cell therapy is target antigen loss or downregulation on malignant cells, measured using flow cytometry [29, 30]. Therefore, changes in antigen expression are important markers of long-term efficacy. Additionally, patients enrolled in CD22-targeting trials may have previously failed CD19 CAR T cell therapy or may have received CD22-targeted antibody therapy. Because these prior therapies could cause antigen loss, we will record antigen expression at baseline as well.
- *Adverse events*: we will evaluate clinical safety of CD22-targeting CAR T cell therapies by reporting the incidence of adverse events. Adverse events are defined as undesired or unintended signs, symptoms, or diagnoses that occur during the study. To qualify as an adverse event, the event must be absent at baseline and/or worsen during the study period. Adverse events of interest will include cytokine release syndrome (CRS), neurotoxicity, infection, and graft-versus-host disease.
  Where available, we will report both the proportion of patients with any CRS and the proportion of patients with severe CRS (grade 3 or higher). Likewise, we will report the proportion of patients with any neurotoxicity and the proportion of patients with severe neurotoxicity (grade 3 or higher).
- *Manufacturing outcomes*: we will extract outcomes related to the manufacturing of CAR T cell products, including events of failure, CAR T cell yields, and transfection efficiency.

#### Tertiary outcomes

If reported, health-related quality of life (HRQoL) or patient-reported outcomes (PRO) will be extracted. HRQoL outcomes are defined as those which measure the “capacity to perform the usual daily activities for a person’s age and major social role” [31]. PROs include any outcome based on data provided by patients themselves. This may include self-reporting on health status, quality of life, treatment satisfaction, etc.

#### Outcome follow-up periods

We will report details of the duration of the treatment response. However, follow-up time points are expected to be highly variable. We will record the length of follow-up.

### Risk of bias (quality) assessment

Most of the studies are expected to be single-arm clinical trials. To assess risk of bias (RoB), we will use the modified Institute of Health Economics tool developed by Grigor et al. for single-arm interventional studies [26]. Studies will be appraised by two reviewers using this tool, with each term given a score of low risk, moderate risk, or high risk of bias.

For each study, RoB terms related to study design (study objectives, design, population, intervention and cointervention, and conflicts of interest) will be appraised using the main study source. If needed, this information may be supplemented by included reports, the clinical trial registry, or through contact with the author. To assess results-related terms (outcome measures, statistical analysis, results, conclusions), the report in which each result is found will be used as the source to appraise its RoB.

Disagreements will be resolved by discussion or by a third-party reviewer. The overall RoB results will be presented as a risk of bias graph.

### Strategy for data synthesis

We will report a narrative synthesis of clinical studies, presenting data in tabular form. A qualitative analysis will include trends across studies regarding efficacy and toxicity, as well as unique findings within studies.

Since we anticipate a small number of existing studies based on a prior informal scoping review, we expect that meta-analysis may be infeasible and/or have limited value. However, we will assess both the number and heterogeneity of studies gathered to determine if meta-analysis is feasible.

In case a meta-analysis is performed, we will report binary outcomes as proportions with 95% confidence intervals. A random effects model (DerSimonia and Laird) will be employed to pool the outcomes. We will use the Cochrane *I*^2^ statistic to assess heterogeneity of effect size in pooled proportions (excluding *N* of 1 study). Considerable heterogeneity will be explored.

The following 4 sections (“Metabias assessment,” “Sensitivity analysis,” “Analysis of subgroups or subsets,” and “Confidence in Cumulative Estimate”*)* will only be applied if a meta-analysis is pursued.

### Metabias assessment

We will use an alternative funnel plot (study size vs. log odds of primary outcome) to assess for publication bias. Reasons for this choice are described previously by Grigor et al. [26].

Publication bias will also be addressed by searching the Clinicaltrials.gov and the ICTRP registries for CAR T-cell trials targeting CD22 in B cell malignancies. Studies in the post-enrollment stage (i.e., active, completed, withdrawn, or terminated) that are not represented in publications will be further examined for possible reasons for lack of publication.

### Sensitivity analysis

If data from conference abstracts are incorporated into a potential meta-analysis, sensitivity analysis will be performed, as suggested by Scherer and Saldanha [32] in order to evaluate whether conference abstracts disproportionately contribute to uncertainty. The sensitivity analysis will be performed by removing data from conference abstracts and evaluating the effect on the results.

### Analysis of subgroups or subsets

Subgroup analysis will depend on the type of data available. A priori subgroups of interest include the following:

- B cell malignancy type (i.e., B-ALL vs. NHL)
- Pediatric/young adults vs. adults
- CD22 CAR T cells alone vs. combination CAR T cells (sequential, dual-targeted CAR, etc.)
- Prior HSCT
- Prior CD19 CAR T cell therapy
- Prior non-CAR T cell immunotherapy

### Confidence in cumulative estimate

If needed, we will apply the GRADE approach to evaluate the confidence in treatment effects [33]. Specifically, the quality of evidence will be appraised based on risk of bias, consistency, directness, precision, and publication bias. A GRADE score of very low, low, moderate, or high will be assigned to indicate the quality of evidence. The quality of evidence reflects the level of confidence of the estimate of treatment effects.

## Discussion

With antigen-negative relapse as an emerging challenge to CAR T cell therapy for B cell malignancies, a vast number of CD22-targeting and multi-target CAR T cell therapies are being explored in clinical trials. A comparison across trials to identify the most effective CAR constructs, methods of multi-targeting, and patient determinants of efficacy are essential to inform clinical use.

The aim of this review is to synthesize evidence on efficacy and safety of CD22 CAR T cells in order to inform future clinical trials. This will also be the first systematic review to collate evidence of different dual-targeting strategies, which will be valuable in assessing the relative efficacy of these various strategies.

Based on an initial scoping review, we found that a large proportion of current phase I data is only reported in conferences and not yet in full-text articles. Therefore, we will be including conference abstracts to maximize the amount of data and to allow for the most up-to-date synthesis of evidence, but we acknowledge that it is lower-quality evidence than full-length articles. As such, we will conduct a sensitivity analysis to account for the decreased rigor of conference abstracts, if a meta-analysis is performed. However, we recognize meta-analysis will likely be inappropriate for the majority of outcomes due to lack of consistent reporting across publications. In this scenario, a narrative synthesis will provide the adequate review of evidence and identification of trends and determinants of efficacy and adverse events.

### Supplementary Information

**Additional file 1:** Search Strategy for MEDLINE, EMBASE, and Cochrane Central Register of Controlled Trials (via Ovid).

## Data Availability

Not applicable.

## Appendix

**Table 1:** Population, intervention, comparators, outcomes, and study characteristic eligibility criteria

Category	Description of criteria
Population	Patients with any B cell malignancy, with no restriction to age or gender, receiving CD22-targeted CAR T cell therapy. B cell malignancies include B cell acute lymphoblastic leukemia (B-ALL), B cell non-Hodgkin lymphoma (B-NHL), and B cell chronic lymphoblastic leukemia (B-CLL).
Intervention	CD22 CAR T cell therapy or any combination CAR regimen that includes a CD22 targeting CAR. This includes examples such as: - Sequential infusion of CD22 CAR T cell with another CAR T cell product - Coadministration of CD22 CAR T cell with another CAR T cell product - Infusion of a CAR T cell population that co-expresses multiple CARs, one being anti-CD22 CAR - Infusion of a CAR T cell population expressing a single multivalent CAR vector that targets CD22 and other antigen(s) Only second-generation CARs and later will be included. First-generation CARs will be excluded as their limited efficacy is well-established [34].
Comparator(s)	- No comparators (single-arm study) - Salvage chemotherapy - Other targeted therapies (ex. other CAR T cell therapies, antibody-based therapies) - Stem cell transplant
Outcome(s)	Primary outcome: - Complete response Secondary outcomes: - Overall response [35] - Minimal residual disease (in B-ALL) - Progressive disease - Relapse - Overall survival - Progression-free survival - B cell aplasia - CAR T cell expansion and persistence - Stem cell transplant post-CAR therapy - Antigen expression (CD19, CD22) on malignant cells before and after CAR T cell therapy - Adverse events (infection, neurotoxicity, cytokine release syndrome, graft-versus-host-disease; other types will be grouped by organ system affected and severity) - Manufacturing outcomes Tertiary outcomes - Health-related quality of life - Patient-reported outcomes
Study characteristics	Interventional: ± controlled, ± randomized

## Abbreviations

B-ALL: B cell acute lymphoblastic leukemia
B-CLL: B cell chronic lymphoblastic leukemia
B-NHL: B cell non-Hodgkin lymphoma
CAR: Chimeric antigen receptor
CR: Complete response
CRS: Cytokine release syndrome
DLBCL: Diffuse large B cell lymphoma
HRQoL: Health-related quality of life
HSL: Health science librarian
HSCT: Hematopoietic stem cell transplant
ICTRP: International Clinical Trials Registry Platform
MRD: Minimal residual disease
PRISMA-P: Preferred Reporting Items for Systematic review and Meta-Analysis Protocols
PRO: Patient-reported outcomes
PROSPERO: International Prospective Register of Systematic Reviews
R/R: Relapsed/refractory
RoB: Risk of bias
