# Supplementary material for: Efficacy and safety of CD22 chimeric antigen receptor (CAR) T cell therapy in patients with B cell malignancies: a protocol for a systematic review and meta-analysis
Source: Syst Rev. 2021 Jan 21;10:35. doi: 10.1186/s13643-021-01588-7 (PMC7819297; doi:10.1186/s13643-021-01588-7)
Supplement: Supplementary file 1 — Additional file 1: Search Strategy for MEDLINE, EMBASE, and Cochrane Central Register of Controlled Trials (via Ovid). [file 13643_2021_1588_MOESM1_ESM.docx]

Search Strategy for MEDLINE, EMBASE, and Cochrane Central Register of Controlled Trials (via Ovid):

1. ((chimeric antigen adj2 receptor*) and (therap* or treat* or immunity or immunotherap* or cell*)).tw,kw.

2. ((car adj3 t adj5 therap*) or (car adj3 t adj5 treat*)).tw,kw.

3. (car adj3 t adj3 immunotherap*).tw,kw.

4. Receptors, Antigen, T-Cell/tu

5. (car therap* or (car adj2 t adj2 cell*)).tw,kw.

6. ((modified or engineered) adj2 (t cell* or t lymphocyte*)).tw,kw.

7. Receptors, Antigen, T-Cell/ and (Adoptive Transfer/ or Immunotherapy, Adoptive/ or Immunotherapy/)

8. car t.tw,kw.

9. or/1-8

10. (cluster adj3 different* adj3 "22").tw.

11. (cd adj3 "22").tw.

12. (cd and "22").kf.

13. CAR22.mp.

14. CART-22.mp.

15. (CD19* and "22").mp.

16. cd22*.mp.

17. (CD20* and "22").mp.

18. 10 or 11 or 12 or 13 or 14 or 15 or 16 or 17

19. 9 and 18

20. 19 use medall

21. ((chimeric antigen adj2 receptor*) and (therap* or treat* or immunity or immunotherap* or cell*)).tw.

22. ((car adj3 t adj5 therap*) or (car adj3 t adj5 treat*)).tw.

23. (car adj3 t adj3 immunotherap*).tw.

24. (car therap* or (car adj2 t adj2 cell*)).tw.

25. ((modified or engineered) adj2 (t cell* or t lymphocyte*)).tw.

26. chimeric antigen receptor/

27. "chimeric antigen receptor T-cell immunotherapy"/

28. car t.tw.

29. or/21-28

30. CD22 antigen/

31. car22.mp.

32. (cd adj3 "22").tw.

33. cd22*.tw.

34. (CD19* and "22").mp.

35. (CD20* and "22").mp.

36. CART-22.mp.

37. or/30-36

38. 29 and 37

39. 38 use oemezd

40. ((chimeric antigen adj2 receptor*) and (therap* or treat* or immunity or immunotherap* or cell*)).tw,kw.

41. ((car adj3 t adj5 therap*) or (car adj3 t adj5 treat*)).tw,kw.

42. (car adj3 t adj3 immunotherap*).tw,kw.

43. Receptors, Antigen, T-Cell/tu

44. (car therap* or (car adj2 t adj2 cell*)).tw,kw.

45. ((modified or engineered) adj2 (t cell* or t lymphocyte*)).tw,kw.

46. Receptors, Antigen, T-Cell/ and (Adoptive Transfer/ or Immunotherapy, Adoptive/ or Immunotherapy/)

47. car t.tw,kw.

48. or/40-47

49. (cluster adj3 different* adj3 "22").tw.

50. (cd adj3 "22").tw.

51. (cd and "22").kf.

52. CAR22.mp.

53. CART-22.mp.

54. (CD19* and "22").mp.

55. cd22*.mp.

56. (CD20* and "22").mp.

57. 49 or 50 or 51 or 52 or 53 or 54 or 55 or 56

58. 48 and 57

59. 58 use cctr

60. 20 or 39 or 59

61. remove duplicates from 60

62. 61 use medall

63. 61 use oemezd

64. 61 use cctr

Search Strategy for Web of Science

1. TS=((chimeric antigen NEAR/1 receptor*) AND (therap* OR treat* OR immunity OR immunotherapy* OR cell*))

2. TS=((car NEAR/2 t NEAR/4 therap*) OR (car NEAR/2 t NEAR/4 treat*))

3. TS=(car NEAR/2 t NEAR/2 immunotherap*)

4. TS=(car therap* OR (car NEAR/1 t NEAR/1 cell*))

5. TS=(((modified OR engineered) NEAR/1 (t cell*)) OR ((modified OR engineered) NEAR/1 (t lymphocyte*)))

6. TS=(car t)

7. #1 OR #2 OR #3 OR #4 OR #5 OR #6

8. TS=(cluster NEAR/1 different* NEAR/2 “22”)

9. TS=(cd NEAR/2 “22”)

10. TS=(CAR22)

11. TS=(CART-22)

12. TS=(CD19* AND "22")

13. TS=(cd22*)

14. TS=(CD20* AND “22”)

15. #8 OR #9 OR #10 OR #11 OR #12 OR #13 OR #14

16. #7 AND #15
